# Effects of different sowing dates on biomass allocation of various organs and allometric growth of *Fagopyrum esculentum*

**DOI:** 10.3389/fpls.2024.1399155

**Published:** 2024-06-07

**Authors:** Heqi Wang, Congwen Wang, Gaohua Fan, Changxing Fu, Yingxin Huang, Xuhe Liu, Shirui Wang, Kunling Wang

**Affiliations:** ^1^ State Key Laboratory of Black Soils Conservation and Utilization, Northeast Institute of Geography and Agroecology, Chinese Academy of Sciences, Changchun, China; ^2^ Jilin Provincial Key Laboratory of Grassland Animal Husbandry, Northeast Institute of Geography and Agroecology, Chinese Academy of Sciences, Changchun, China; ^3^ University of Chinese Academy of Sciences, Beijing, China; ^4^ State key Laboratory of Vegetation and Environmental Change, Institute of Botany, Chinese Academy of Sciences (CAS), Beijing, China; ^5^ College of Life Sciences, Jilin Agricultural University, Changchun, China

**Keywords:** sowing dates, reproduction, biomass allocation, allometric growth, *Fagopyrum esculentum*

## Abstract

**Introduction:**

The sowing date plays a crucial role in influencing the growth and reproduction of plants, with its specific impact on biomass allocation and allometric growth remaining unclear. Understanding these effects is essential for optimizing agricultural practices and enhancing crop productivity.

**Methods:**

To investigate the effects of sowing dates on biomass allocation and allometric growth, a field experiment was conducted with sequential sowings of Fagopyrum esculentum from April 12th to August 11th in 2018. Biomass measurements were taken across various plant organs, and corresponding allocation calculations were made. A detailed analysis of the allometric growth relationship involving organ biomass variations was performed.

**Results:**

The study revealed that the accumulation and allocation of organ biomass in buckwheat were significantly impacted by the sowing dates. Delayed planting led to reduced vegetative growth and increased biomass allocation towards reproduction. Allometric parameters such as exponent, constant, and individual size of buckwheat were notably affected by delayed planting. Interestingly, the allometric exponents governing the relationships between reproductive vs. vegetative biomass and belowground vs. aboveground biomass exhibited varying trends across different sowing dates.

**Discussion:**

Notably, late sowings resulted in significantly higher reproductive biomass compared to early and middle sowings. These findings highlight the nuanced relationship between plant size and reproductive biomass under different sowing dates, emphasizing the critical role of planting timing in shaping mature plant sizes and reproductive outcomes. The study underscores the importance of considering sowing dates in agricultural practices to optimize plant growth and productivity.

## 1 Introduction

Plants undergo growth and reproduce throughout their life cycle. As plants experience grow, they need to adapt the dynamic environmental conditions. Previous research indicates that the increasing impact of climate change has repercussions on plant phenology, consequently influencing their growth and reproductive processes (Piao et al., 2019). Consequently, plants need to harmonize resource allocation among different organs and adjust their traits to adapt to the changing environment (Poorter et al., 2012; Heilmeier, 2019; Myers-Smith et al., 2019).

Biomass allocation significantly influences plant growth and productivity (McCarthy and Enquist, 2007). Plants continually allocate acquired resources, such as carbon and nutrients, to different tissues during growth (Shipley and Meziane, 2002). How plants respond to resource availability is a central question in plant ecology (McConnaughay and Coleman, 1999). The applications of biomass allocation in agriculture are extensive, as allocation patterns and their degree of variation between species constrain biomass production and utilization (Poorter et al., 2012). In essence, biomass allocation may vary over time, across environments, and among species (Niklas, 1994; Reich, 2002). In agroecosystems, crop productivity depends not only on the accumulation of dry matter, but also on the efficient distribution of dry matter to economically important plant parts (Kumar et al., 2006). Nutrition, temperature, solar radiation, and water conditions also affect biomass allocation (Kage et al., 2004; Poorter et al., 2012; Steinfort et al., 2017; Lizaso et al., 2018). Thus, partitioning patterns between the shoots and roots change with the sowing date and environmental conditions (Justes et al., 2002; Bonelli et al., 2016). In durum wheat (*Triticum turgidum*), late sowing resulted in a higher grain protein concentration than normal sowing, partially compensating for the reduction in dry matter accumulation (Ferrise et al., 2010). For example, the delayed planting of maize (*Zea mays*) and soybean (*Glycine max*) limited their photosynthetic source capacities, resulting in low-level light interception, reduced translocation of photo-assimilates to different organs, and decreased yields (Bonelli et al., 2016). Small differences in arrival time influence composition and productivity of plant communities (Koerner et al., 2008).

Various plant organs indicate different physiological and ecological functions. Throughout the process of plant growth and reproduction, all the organs exhibit conspicuous allometric growth, and this is an inherent characteristic determined by species inheritance (Weiner, 2004; Huang et al., 2009). Viewed from an allometric perspective, allocation is regarded as a size-dependent process; allometry constitutes the quantitative relationship between growth and allocation. Therefore, most studies about allocation should be posed allometrically, rather than as mere ratios or proportions. Plants evolve allometric patterns in response to numerous selection pressures and constraints, elucidating various behaviors among plant populations (Weiner, 2004). As an important aspect of plant ecology research, allometric growth analysis serves as a crucial method for studying plant adaptation strategies. The inherent relationship between plant density, individual size, and traits can be systematically elucidated through allometric growth analysis. This approach facilitates the analysis of individual size and reveals the strategic adjustments that plants make in response to abiotic factors. Several studies have demonstrated that the allometric growth of plants is significantly influenced by their reproductive period, particularly in relation to the relationship between the reproductive organs and individual size (Gomez-Fernandez and Milla, 2022). Therefore, it is necessary to take into account the changes in reproductive periods. Furthermore, the results derived from the allometric growth analysis of a single sample lack rigor; multiple rounds of sampling should be conducted throughout the entire plant life cycle to reveal the allometric growth patterns of different plant organs (Weiner, 2004).

The modulation of plant plasticity in natural environments is significantly influenced by abiotic factors and growth stages (Wang and Zhou, 2022). The emergence time, a mechanism intricately linked to environmental conditions, is considered a precise determinant of habitat selection or niche construction, shaping the plant’s interaction with the environment (Donohue, 2003, 2005). Consequently, it plays a crucial role in seedling establishment and subsequent growth (Donohue, 2005; Wilczek et al., 2009; Burghardt et al., 2015), particularly in competitive environments (Kelly and Levin, 1997; Dyer et al., 2000; Seiwa, 2000).

*Fagopyrum esculentum*, an annual herb within the *Polygonaceae* family, exhibits distinctive features. Buckwheat is widely cultivated worldwide, including in countries such as China, Russia, and the United States, and can be biannual sown in some region and boasts rapid grow. Characterized by white flowers and an infinite inflorescence, it undergoes a 30 to 45-day transition from flowering to maturity. Seed harvesting occurs when the plant’s seeds reach 75% to 80% maturity, marked by a brown hue resulting from uneven maturation. The plant is sensitive to unfavorable environmental factors, especially to frost, extreme temperatures, and droughts (Cogoni et al., 2013). Due to its inherent tolerance to various abiotic stresses and a short life cycle, buckwheat has garnered attention as a model crop plant (Zargar et al., 2023). It is primarily cultivated for its seeds’ advantageous chemical composition, which includes high levels of starch, minerals, vitamins, rutin, antioxidants, and dietary fiber. Additionally, its unique amino acid composition distinguishes it as particularly abundant in lysine and arginine while being gluten-free (Plazek et al., 2023). Meanwhile, it has economic value because of its seed yield. For example, The demand for buckwheat, particularly organic buckwheat, has witnessed a rapid surge in recent years and is predominantly fulfilled through imports from non-EU countries, notably China. Buckwheat generally exhibits a modest grain yield. However, it has demonstrated promising potential for commercial cultivation in Europe with grain yields ranging from 1.2 to 3.0 t ha^−1^ (Domingos and Bilsborrow, 2021).

However, there remains a dearth of research on the effects of different sowing dates on the biomass allocation of various organs and the allometric growth of *F. esculentum*. We conducted a field experiment involving the sowing of *F. esculentum* on nine distinct dates, intentionally inducing delayed planting. Our objective was to examine the biomass allocation of different organs and allometric growth within natural environments. Specifically, we aimed to address the following questions: (1) What impact does delayed planting affect biomass allocation and accumulation? (2) How does the planting time influence the allometric growth of *F. esculentum*?

## 2 Materials and methods

### 2.1 Experimental design

This experiment was conducted in 2018 at the Changling Ecological Research Station for Grassland Farming (ERSGF), Chinese Academy of Sciences (44°33′N, 123°31′E, 145 m alt), in the southern part of the Songnen grassland of northeast China. This region is characterized by a dry and windy spring and a hot and wet summer, with an average annual air temperature of 4.6–6.4°C, and average annual precipitation and evaporation totals of 410 mm and 1000–2000 mm, respectively. The average air temperature peaks in July (>20°C) during the primary growth season (between June and August), and precipitation during this period contributes 70%– 80% of the total annual precipitation. The annal average sunshine duration is 2505.53 hours (**Supplementary Table S1**). The original soil in the experimental field (aeolian sandy soil; pH = 8.61 ± 0.05; EC (soil electric conductivity) = 0.26 ± 0.02) was low in nutrients (SOM (soil organic matter) = 3.04 ± 0.46 g kg^−1^; available N = 31.58 ± 3.08 mg kg^-1^; available P = 30.69 ± 1.51 mg kg^-1^; available K = 162.86 ± 11.49 mg kg^-1^) at the 0–10 cm soil depth during the growth season (**Supplementary Table S2**).

The variety we used is Jiqiao 10. It is a new buckwheat variety grown by mixed selection method. Its main advantages are high quality, high yield and stress resistance. Seeds of Jiqiao 10 were purchased from the ChunYu Seed Company (Jilin, China). The seeds were stored in dark at room temperature (18–20°C) before sowing.

A completely randomized design was implemented with four replicates, and the area of the individual plots totaled approximately 32 m^2^. (There were 7 ridges per plot; the ridge distance was 65 cm, and the ridge length was 7 m). In this experiment, we sowed the plants on the following nine dates: 12 April, 27 April, 12 May, 27 May, 11 June, 26 June, 11 July, 26 July, and 11 August (**Table 1**). Thus, there were 36 plots in total. In addition, the growing conditions, such as, accumulated temperature (°C), effective accumulated temperatures (°C), and the precipitation values (mm) were changed among the sowing treatments (**Supplementary Table S3**). In each plot, we sowed 941 g of seeds. The sowing dates of buckwheat is generally sown from late May to early June in Northern China. The amounts of actual NPK fertilizer applied in each plot were 22.5 kg ha^-1^, 3.86 kg ha^-1^, and 8.67 kg ha^-1^, respectively. All the plots were irrigated immediately after sowing, and also irrigated during the dry season in order to prevent drought stress. After seedling emergence, the seedlings were thinned by 1 million plants per hectare when they reached the four-leaf stage to ensure uniform initial density across all sowing treatments. Undesired weeds were controlled adequately through manual weeding at regular intervals.

**Table 1:** Information about sowing dates, symbols, groups, growth times, and the growth of maximum reproductive biomass in *F. esculentum*.

Sowing dates	Symbol	Group	Growth time (days)	The growth time of Maximum reproductive biomass (days)
12 April	SD1	Early	181	118
27 April	SD2	Early	166	97
12 May	SD3	Early	151	104
27 May	SD4	Middle	136	89
11 June	SD5	Middle	121	86
26 June	SD6	Middle	106	106
11 July	SD7	Late	91	91
26 July	SD8	Late	76	76
11 August	SD9	Late	60	60

### 2.2 Plant sampling and measurements

Plant biomass accumulation and allocation were measured at various sampling times. We selected eight plants in each plot with four replicates for each treatment from 11 June to 10 October. The plant organs were divided into five parts, including the root, stem, leaf, flower, and mature seed. The value of biomass per organ was obtained after oven drying the samples at 65°C for 48 h. The total biomass of the plant represents the sum of the biomass from five organs. The vegetative biomass represents the sum of the stem, leaf, and root biomass. The reproductive biomass represents the sum of the flower and mature seed biomass. The aboveground biomass represents the sum of stem, leaf, flower, and mature seed biomass. The belowground biomass represents the root biomass. The aboveground, belowground, reproductive, and vegetative biomass allocations were all obtained by dividing their respective biomass by the total biomass.

### 2.3 Data analysis

Firstly, the reproductive biomass accumulation was analyzed for all the sowing dates (SD1-SD9) throughout sampling (**Supplementary Figure S1**). Then, the maximum reproductive biomass of all the plants was selected to analyze the differences in biomass accumulation and allocation among various organs (vegetative organs and reproductive organs) of *F. esculentum* using one-way analysis of variance (ANOVA). We performed *post hoc* tests using LSD. According to the significant difference in the total biomasses when the maximum reproductive biomass was reached in each sowing period, the plants were divided into three groups (early, middle, and late sowing treatments) (**Table 1** and **Supplementary Table S4**). Locally weighted regression was used to fit the variation in reproductive, vegetative, belowground, and aboveground biomass with the delay in the sowing date and growth time. Standardized major axis regression was used to analysis the dynamic and static allometric growth among the different biomass of the organs (reproductive biomass vs. vegetative biomass; belowground biomass vs. aboveground biomass) of *F. esculentum* using Smatr package in R Version 4.2.2 (R Core Team, 2022). Standardized major axis regression is widely used to estimate the relationship between two variables for line-fitting in allometry (Warton et al., 2006). The allometric relationship between X and Y was Y=bX^a^, where a was the allometric exponent (slope) and b was the allometric coefficient (constant) or “scaling factor” (Y intercept). After logarithmic transformation, the equation is logY = a+ blogX (Huang et al., 2009). The dynamic and static allometric exponents and constants of reproductive biomass vs. vegetative biomass and belowground biomass vs. aboveground biomass from the early to late sowing dates were analyzed. All the statistical analyses and plots were performed and drawn in R Version 4.2.2 (R Core Team, 2022).

## 3 Results

### 3.1 The effects of sowing dates on biomass accumulation and allocation

**Figure 1:**
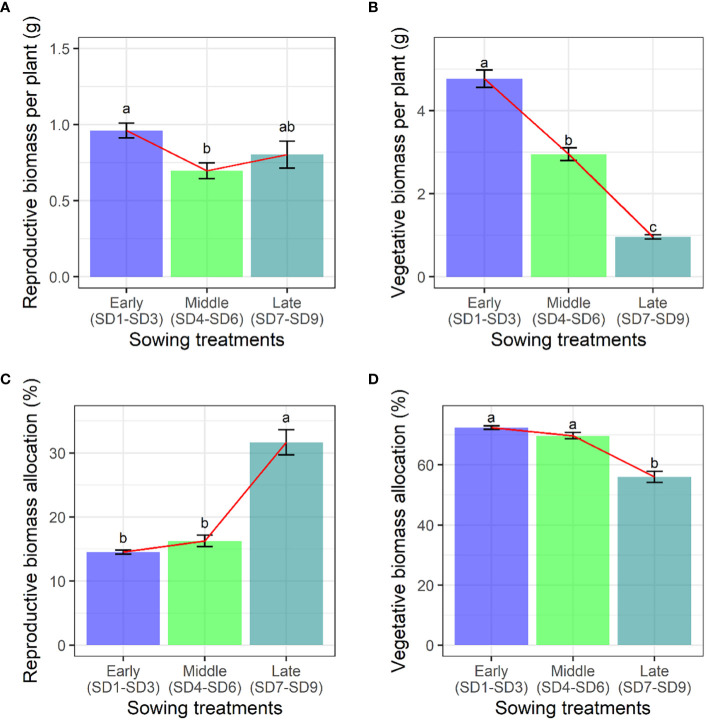
Effects of sowing treatments (early, middle, and late) on **(A)** reproductive biomass, **(B)** vegetative biomass, **(C)** reproductive biomass allocation, and **(D)** vegetative biomass allocation when *F. esculentum* reached the maximum reproductive biomass. Different lowercase letters represent significant differences among three sowing treatments at *p*<0.05.

Firstly, the impact of the sowing date on the biomass accumulation of *F. esculentum* was examined. The sowing dates had a significant effect on the reproductive, vegetative, aboveground, and belowground biomass of *F. esculentum*, thereby impacting the maximum reproductive biomass (*p* < 0.001). Specifically, with the delay in the sowing date, the reproductive biomass of *F. esculentum* significantly decreased from early to middle sowing treatments. However, the reproductive biomass did not exhibit any statistically significant variation among late sowing, early sowing, and middle sowing (**Figure 1A**). The vegetative biomass of *F. esculentum* increased gradually with a delayed germination time (**Figure 1B**). Considering the reproductive and vegetative biomass allocation of *F. esculentum* on the maximum reproductive biomass, our findings demonstrate that the reproductive biomass allocation value remained 14%~16% for the early and middle sowing treatments, and then increased to 32% for the late sowing treatment (**Figure 1C**). When we compared the vegetative biomass allocation, we found that they remained at 70%-72% for the early and middle sowing treatments, and then decreased to 56% (SD7-SD9) (**Figure 1D**). Similarly, the allocation and accumulation of reproductive and vegetative biomass in all the sowing treatments (SD1-SD9) were in line with the above findings (**Supplementary Tables S4**, **S5**).

**Figure 2:**
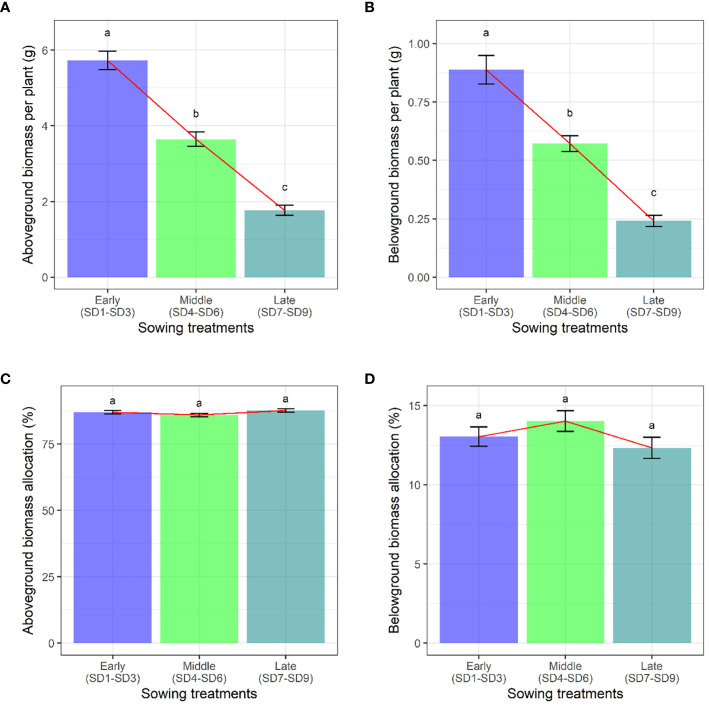
Effects of sowing treatments (early, middle, and late) on **(A)** aboveground biomass, **(B)** belowground biomass, **(C)** aboveground biomass allocation, and **(D)** belowground biomass allocation when *F. esculentum* reached the maximum reproductive biomass. Different lowercase letters represent significant differences among three sowing treatments at *p*<0.05.

When we compared the aboveground and belowground biomass of *F. esculentum*, we found that they decreased gradually with the delay in the sowing date (**Figures 2A, B**). However, the sowing treatments had no significant effect on biomass allocation (**Figures 2C, D**). With the delay of planting time (SD1-SD9), The aboveground biomass and belowground biomass were gradually decreased. However, the aboveground biomass and belowground biomass allocation were not significantly different among sowing treatments (SD1-SD9) (**Supplementary Tables S4**, **S5**).

**Figure 3:**
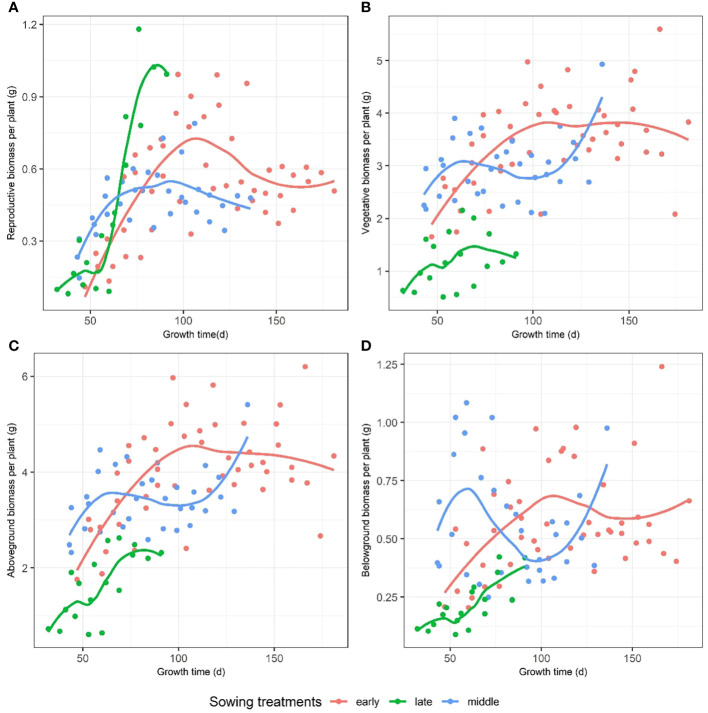
The dynamic changes in **(A)** reproductive, **(B)** vegetative, **(C)** aboveground, and **(D)** belowground biomasses with growth time among early, middle, and late sowing treatments on *F. esculentum*.

We compared the reproductive, vegetative, aboveground, and belowground biomasses with the growth time among the early, middle, and late sowing groups. The early and middle sowing treatments caused an increased accumulation of vegetative and reproductive biomasses compared to that of the late sowing treatment (**Figure 3**), but the latter caused the plants to reach the maximum reproductive biomass in a shorter period of time, which was significantly higher than those of the middle and late sowing treatments (**Figure 3A**). In terms of the aboveground and belowground biomass, the early and middle sowing treatments caused an increased accumulation of biomass compared to that of the late sowing treatment (**Figures 3B–D**).

### 3.2 Allometric growth of different organs

**Figure 4:**
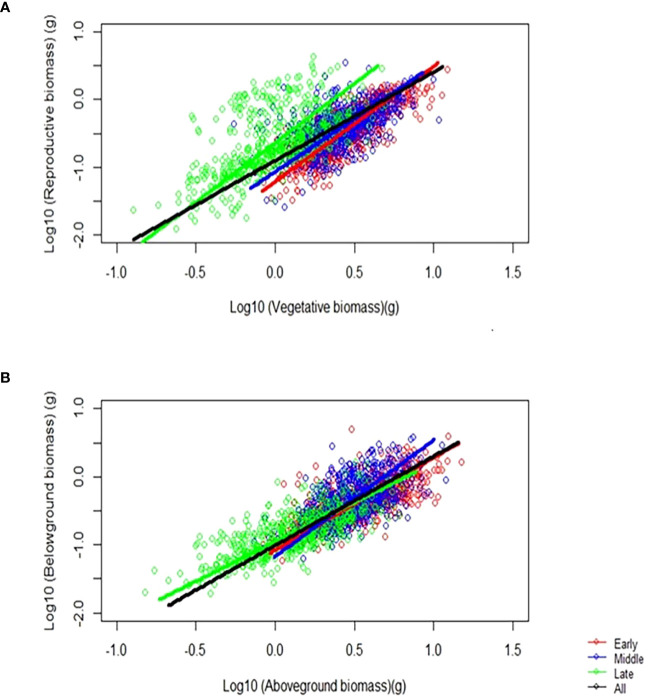
The effect of sowing dates on the **(A)** allometric relationship between reproductive and vegetative biomass, and **(B)** belowground and aboveground biomass among the early, middle, and late sowing treatments when the maximum reproductive biomass of *F*. *esculentum* was reached.

By analyzing the allometric growth of the organ biomass of *F. esculentum*, we found a power function growth pattern among the different sowing dates. When the data transformed by log10, we can figure out the allometric relationship between reproductive biomass vs. vegetative biomass, and belowground biomass vs. aboveground biomass among the early, middle, and late sowing treatments (**Figure 4**). The allometric growth of the belowground vs. aboveground biomass and reproductive vs. vegetative biomass changed over time (*p*< 0.05). We found that the allometric exponent and constant were significantly changed by the sowing treatments (*p* < 0.05), of which the homogeneity of the slope and shifts in the intercept and along the common slope were significantly different among the sowing treatments (early, middle and late) (**Supplementary Table S3**). Sowing on different dates had a significant effect on the allometric exponent and constant and individual size of *F. esculentum* (*p* < 0.05) (**Table 2**).

**Table 2:** The allometric exponent and constant of reproductive vs. vegetative biomass and below-ground vs. aboveground biomass of *F. esculentum* among the sowing dates.

Sowing treatments	Traits	Allometric equation	Allometric exponent	Allometric constant	*R* ^2^	*p*
Early	Belowground vs. Aboveground	Y=1.358X-1.077	1.358	-1.077	0.502	<0.001
Middle	Belowground vs. Aboveground	Y=1.712X-1.170	1.712	-1.170	0.373	<0.001
Late	Belowground vs. Aboveground	Y=1.158X-0.955	1.158	-0.955	0.555	<0.001
All	Belowground vs. Aboveground	Y=1.311X-1.006	1.311	-1.006	0.610	<0.001
Early	Reproductive vs. Vegetative	Y=1.702X-1.212	1.702	-1.212	0.651	<0.001
Middle	Reproductive vs. Vegetative	Y=1.572X-1.065	1.572	-1.065	0.332	<0.001
Late	Reproductive vs. Vegetative	Y=1.752X-0.635	1.752	-0.635	0.354	<0.001
All	Reproductive vs. Vegetative	Y=1.306X-0.901	1.306	-0.901	0.413	<0.001

The symbol Y of allometric equation represents belowground biomass and reproductive biomass. The symbol X of allometric equation represents aboveground biomass and vegetative biomass. The Y and X of the allometric equation were transformed by log10. Early (SD1-SD3); Middle (SD4-SD6); and Late (SD7-SD9).

**Figure 5:**
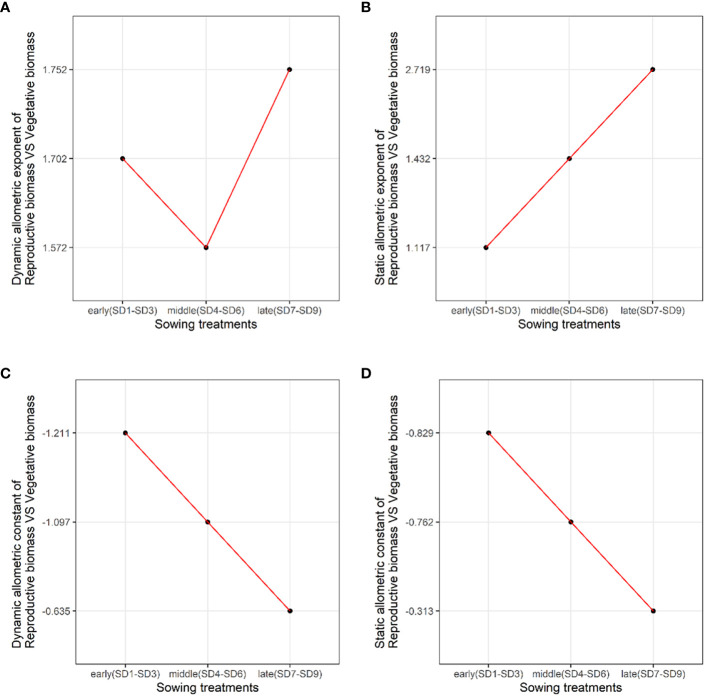
The changes in the dynamic **(A)** allometric exponent, **(B)** static allometric exponent, **(C)** dynamic allometric constant, and **(D)** static allometric constant of reproductive vs. vegetative biomass among the early, middle, and late sowing dates when the maximum reproductive biomass of *F*. *esculentum* was reached.

**Figure 6:**
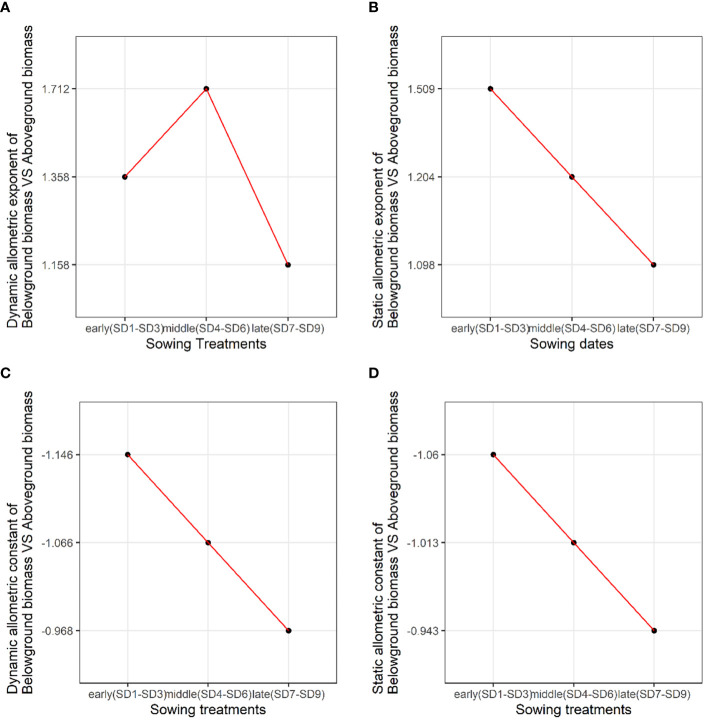
The changes in the **(A)** dynamic allometric exponent, **(B)** static allometric exponent, **(C)** dynamic allometric constant, and **(D)** static allometric constant of belowground vs. aboveground biomass among the early, middle, and late sowing dates when the maximum reproductive biomass of *F*. *esculentum* was reached.

We analyzed the allometric exponent and constant (dynamic and static) of the reproductive vs. vegetative biomass and belowground vs. aboveground biomass among the early, middle, and late sowing treatments. The dynamic allometric exponent and constant reflect the slope and intercept of the allometric growth equation between the sampling initiation and the period when plants reach their maximum reproductive biomass (reproductive biomass vs. vegetative biomass, belowground biomass vs. aboveground biomass).Static allometric exponent and constant reflect the slope and intercept of the allometric growth equation at the point when plants reach their maximum reproductive biomass (reproductive biomass vs. vegetative biomass, belowground biomass vs. aboveground biomass). With the delay in planting time, the dynamic allometric exponent of the reproductive vs. vegetative biomass first decreased from the early to middle sowing treatments, and then increased or the late sowing treatment (**Figure 5A**). However, the static allometric exponent of the reproductive vs. vegetative biomass increased gradually from the early to late sowing treatments (**Figure 5B**). As for the dynamic and static allometric exponents of the belowground vs. aboveground biomass, we found the former first increased, and then gradually decreased from the early to late sowing treatments; on the contrary, the static allometric exponent decreased gradually with the delay in planting time (**Figures 6A, B**). In general, the allometric exponents (dynamic and static) between the reproductive vs. vegetative biomass and belowground vs. aboveground biomass had an opposite tendency. It suggested that the trade-off between reproductive vs. vegetative and belowground vs. aboveground growth. The dynamic and static allometric constants decreased gradually with the delay in sowing time between the reproductive vs. vegetative biomass and belowground vs. aboveground biomass (**Figures 5C, D**, **6C, D**).

## 4 Discussion

### 4.1 The effects of sowing dates on biomass accumulation and allocation

Buckwheat is an important economic crop with rich nutritional content in its seeds, possessing extremely high economic value. Understanding the pattern of resource allocation in buckwheat is the primary motivation for studying the changes in plant growth patterns. Environmental changes exert varying degrees of influence on the biomass allocation of different organs. Sowing on different dates produced a plastic phenological response, impacting the key life cycle stages, including the onset of reproduction (Zhou et al., 2005). Our findings reveal that early sowing tends to allocate more biomass to the vegetative organs, such as the leaves and stems (**Figures 1**, **2**). This can be attributed to the survival strategy of the plant (Zhou et al., 2005). Conversely, late sowing of *F. esculentum* leads to a higher allocation of biomass to reproductive organs rather than vegetative ones, facilitating rapid growth. As the sowing date is delayed, the size (vegetative or aboveground biomass) of *F. esculentum* is gradually decreases. Biomass accumulation and allocation to different components also showed plastic variation based on sowing dates (**Supplementary Tables S4**, **S5**). Earlier sowing resulted in heavier root, stem, leaf, and reproductive tissues, providing ample time and resources for both vegetative and reproductive growth. Earlier-sown plants allocated more biomass to the roots and stems, while less was directed to the reproductive tissues and leaves compared with the later-germinating plants. Accumulation and partitioning of biomass in reproductive organs play crucial roles in plants’ fitness across diverse environments (Weiner, 1988; Hartnett, 1990; Sadras et al., 1997; Vega et al., 2000). The contrasting reproductive patterns between the early and late germinating plants indicate varied fitness priorities in the different environments. In unfavorable conditions, a shorter life cycle, flowering earlier, and enhanced reproductive allocation confer advantages, while in favorable conditions, plants tend to have a longer lifespan, delayed flowering, and extended vegetative growth with a great reproductive output to enhance fitness (Sultan, 2000). The greater investment of resources in reproduction during a short life cycle is characteristic of annual weeds, where fewer resources are allocated to the supporting structures like roots and stems. This flexible partitioning appears to be typical behavior observed in annual plants (Hermanutz and Weaver, 1996; Cheplick, 2001). Wang and Zhou (2022) conducted an experiment on the effect of emergence time and population density on the dynamic reproductive plasticity of *Abutilon theophrasti*, and found that both advanced and delayed emergence resulted in less vegetative growth and earlier reproduction (Wang and Zhou, 2022). Moreover, environmental factors may affect the optimal relative allocation for survival, growth, and reproduction, shaping cost functions for individual fitness components (Sletvold and Agren, 2015). *F. esculentum*, being a short-day plants, exhibits photoperiodic control as the primary environmental cue for the flowering in short-lived herbs (Rathcke and Lacey, 1985; Klinkhamer et al., 1987; Kudoh et al., 1995). Vegetative growth switches to reproductive growth with decreasing day-length (Huang et al., 2000). With the delay in sowing, the day length gradually became shorter, so *F. esculentum* reproduced earlier. This plant demonstrates the ability to regulate its life cycle in response to environmental changes. A plant’s ability to reproduce early can be considered as an opportunity to successfully complete its life history in unfavorable conditions, as is the case with agricultural weeds that can set seeds before crop harvesting.

However, our study revealed that the reproductive biomass does not exhibit a negative linear relationship with the size of individuals, challenging previous findings suggesting a decrease in reproductive biomass with size (Weiner et al., 2009). Previous study has shown that the populations of *F. esculentum* sown at higher densities supported significantly more biomass per unit volume for a given canopy height (Li et al., 2013). Unlike prior research indicating size-dependent reproductive biomass reduction, our results demonstrated that reproductive biomass is not size-independent. As sowing date is delayed, total biomass gradually decreased, however, initially decreases from early to mid-sowing dates and subsequently increases from mid to late sowing dates. Research has shown that the relationship between plant size and reproductive output is pivotal for a plant’s strategy in converting growth into fitness (Weiner et al., 2009). They propose that a plant, with specific resource amounts at any given time, allocates these resources to different tissues. Reproduction is commonly gauged by the reproductive biomass divided by total biomass, assuming the reproductive effort ratio is size-independent, but our findings indicate a size-dependent reproductive allocation. Furthermore, the plant size is determined by numerous interacting factors (Li et al., 2015). Several studies assert that the growth duration influences plant development, and the growth dynamics are highly functionally coordinated with the plant size (Gomez-Fernandez and Milla, 2022). Our results suggest that the sowing treatments influence the maximum reproductive biomass. Reproductive, vegetative, aboveground, and belowground biomasses changed over time. However, the impact of sowing treatments on *F. esculentum* biomass varied over time (**Figure 3**). When categorizing sowing dates into early, middle, and late treatments, the reproductive biomass of *F. esculentum* initially increased, and then gradually decreased over time for early and mid-stage seeding. Conversely, for the plants sown late, reproductive biomass initially increased, and then decreased over time, with their maximum reproductive biomass substantially higher than that of the plants sown at the early and middle (**Figure 3**).

### 4.2 The effects of sowing dates on allometric growth

Allometric growth analysis, as a crucial method in plant ecology research, facilitates the systematic elucidation of the inherent relationship between plant density, individual size, and traits. It enables the analysis of individual size and reveals the strategic adjustments made by plants make in response to biotic and abiotic factors. Our study revealed that sowing *F. esculentum* at different times significant influenced the allometric trend, altering the allometric exponent and constant (**Figures 5**, **6**). Similarly, previous study has found that the allometric analysis of reproductive and vegetative biomass across treatments showed an increase in reproductive effort with varying planting densities, but decrease when the plant were sown on different dates. The allometric exponent between the treatments varied significantly with the sowing date (Wang et al., 2006). However, our study has demonstrated opposite tendencies in the dynamic and static allometric exponents between the reproductive vs. vegetative biomass and belowground vs. aboveground biomass (**Figures 5**, **6**). This suggests that the sowing treatments experience trade-offs between reproductive vs. vegetative biomass and belowground vs. aboveground biomass. Although the dynamic allometric exponent of reproductive vs. vegetative biomass was the highest among the sowing treatments, that of the belowground vs. aboveground biomass was the lowest. In summary, analyzing the allometric relationship allows us to discern resource partitioning in a plant sown late.

The size and yield of plants are intricately linked to their ontogeny, particularly the growth dynamics (Gomez-Fernandez and Milla, 2022). Our data collection spanned from the reproduction to the end of growth stages, overlooking the periods from emergence to reproduction. To comprehensively explore the effects of sowing on different dates, future studies should consider the growth dynamics throughout the plant’s entire life history. Meanwhile, we designed a field experiment to determine the suitable sowing dates in the northeast area of China. However, our study still has some limitations; we did not study *F. esculentum* throughout its whole life history.

## 5 Conclusions

*F. esculentum*, an annual herb within the *Polygonaceae* family, exhibits distinctive features. Buckwheat is widely cultivated worldwide, including in countries such as China, Russia, and the United States, and can be biannual sown and boasts rapid grow. Our results demonstrate that the biomass allocation and allometric growth (reproductive biomass vs. vegetative biomass, belowground biomass vs. aboveground biomass) of *F. esculentum* are significantly affected by the sowing date. Delayed sowing leads to an increase in the reproductive biomass allocation of *F. esculentum*. Furthermore, delayed planting had significant effects on the allometric exponent, constant, and individual size of *F. esculentum*. In general, the allometric exponents (dynamic and static) between the reproductive vs. vegetative biomass and belowground vs. aboveground biomass exhibit an opposite tendency. Our study reveals that the reproductive biomass does not exhibit a negative linear relationship with the size of the individuals. In summary, our results contribute to a more comprehensive understanding of the effect of sowing dates on plants’ reproductive biomass, and highlight the role of emergence time among mature plants with diverse sizes and reproductive biomass. It is crucial to extend study on this plants’ adaptations from emergence to maturity across various environments.

## Data Availability

The original contributions presented in the study are included in the article/**Supplementary Material**. Further inquiries can be directed to the corresponding author.
