# Corrigendum: Effects of different sowing dates on biomass allocation of various organs and allometric growth of *Fagopyrum esculentum*

**DOI:** 10.3389/fpls.2024.1525982

**Published:** 2024-12-05

**Authors:** Heqi Wang, Congwen Wang, Gaohua Fan, Changxing Fu, Yingxin Huang, Xuhe Liu, Shirui Wang, Kunling Wang

**Affiliations:** ^1^ State Key Laboratory of Black Soils Conservation and Utilization, Northeast Institute of Geography and Agroecology, Chinese Academy of Sciences, Changchun, China; ^2^ Jilin Provincial Key Laboratory of Grassland Animal Husbandry, Northeast Institute of Geography and Agroecology, Chinese Academy of Sciences, Changchun, China; ^3^ University of Chinese Academy of Sciences, Beijing, China; ^4^ State key Laboratory of Vegetation and Environmental Change, Institute of Botany, Chinese Academy of Sciences (CAS), Beijing, China; ^5^ College of Life Sciences, Jilin Agricultural University, Changchun, China

**Keywords:** sowing dates, reproduction, biomass allocation, allometric growth, *Fagopyrum esculentum*

In the published article, there was an error in the **Funding** statement: The funding statement for the “Strategic Priority Research Program of the Chinese Academy of Sciences (XDA28110202)”. The correct **Funding** statement appears below.

The correct statement is “This study was funded by the Strategic Priority Research Program of the Chinese Academy of Sciences (XDA28110201) and Major Science and Technology Programs in Jilin Province (20230303008SF).”

The authors apologize for this error and state that this does not change the scientific conclusions of the article in any way. The original article has been updated.

