# Supplementary material for: Effects of different sowing dates on biomass allocation of various organs and allometric growth of Fagopyrum esculentum
Source: Front Plant Sci. 2024 Jun 7;15:1399155. doi: 10.3389/fpls.2024.1399155 (PMC11190297; doi:10.3389/fpls.2024.1399155)
Supplement: Supplementary file 1 [file DataSheet1.docx]

**Supplementary Materials:**

**Table S1.** Study site information for latitude (°N), longitude (°E), mean annual temperature (MAT, °C), mean annual precipitation (MAP, mm), and annual average sunshine (h).

| Latitude (°N) | Longitude (°E) | MAT (°C) | MAP (mm) | Annual average sunshine（h） |
| --- | --- | --- | --- | --- |
| 44°33′ | 123°31′ | 4.6-6.4 | 410 | 2505.53 |

**Table S2.** Soil properties of the experimental sampling site.

| Soil type | Soil depth  (cm) | pH | Soil electric conductivity (EC)(ms cm^-1^) | Soil organic matter (SOM)  (g kg^-1^) | Available N  (AN)  (mg kg^-1^) | Available P  (AP)  (mg kg^-1^) | Available K  (AK)  (mg kg^-1^) |
| --- | --- | --- | --- | --- | --- | --- | --- |
| Aeolian  sandy soil | 0-10 cm | 8.61±0.05a | 0.26±0.02a | 3.04±0.46a | 31.58±3.08a | 30.69±1.51a | 162.86±11.49a |
|  | 10-20 cm | 8.67±0.08a | 0.21±0.01b | 3.24±0.37a | 25.43±1.90b | 24.64±2.62b | 124.56±5.38b |

**Table S3.** Study site information for accumulated active temperature (℃), sum of effective accumulated temperature (℃), and sum of precipitation (mm) during the growth time among sowing dates.

| Sowing dates | Accumulated active temperature (℃) | Sum of effective accumulated temperature (℃) | Sum of precipitation (mm) |
| --- | --- | --- | --- |
| SD1 | 3460.944 | 1791.778 | 396 |
| SD2 | 3261.833 | 1732.278 | 390.5 |
| SD3 | 3024.278 | 1644.722 | 390.5 |
| SD4 | 2737.222 | 1507.222 | 374.1 |
| SD5 | 2406.667 | 1326.667 | 349.1 |
| SD6 | 2076.611 | 1146.611 | 265.3 |
| SD7 | 1715.889 | 935.8889 | 175.4 |
| SD8 | 1479.556 | 721.2778 | 152.6 |
| SD9 | 1065.611 | 467.3333 | 143.2 |

**Table S4.** Effects of sowing date on biomass of different organs of *F. esculentum.*

| **Sowing dates** | | **Total biomass (g)** | **Aboveground biomass (g)** | **Belowground biomass (g)** | **Vegetative biomass (g)** | **Reproductive biomass (g)** |
| --- | --- | --- | --- | --- | --- | --- |
| SD1 | **2018/4/12** | 6.671±0.475a | 5.817±0.418a | 0.855±0.093a | 5.681±0.413a | 0.991±0.073abc |
| SD2 | **2018/4/27** | 6.941±0.363a | 5.968±0.313a | 0.973±0.108a | 5.948±0.304a | 0.993±0.075abc |
| SD3 | **2018/5/12** | 6.252±0.610a | 5.414±0.533a | 0.838±0.118a | 5.350±0.517a | 0.902±0.101abc |
| SD4 | **2018/5/27** | 4.732±0.426b | 4.197±0.392b | 0.535±0.052bc | 4.004±0.355b | 0.728±0.076cd |
| SD5 | **2018/6/11** | 4.443±0.302bc | 3.838±0.286b | 0.605±0.068b | 3.869±0.240b | 0.574±0.071d |
| SD6 | **2018/6/26** | 3.461±0.282cd | 2.887±0.240c | 0.574±0.058b | 2.672±0.208c | 0.789±0.117bcd |
| SD7 | **2018/7/11** | 2.437±0.239d | 2.200±0.215c | 0.237±0.041de | 1.414±0.130d | 1.023±0.130ab |
| SD8 | **2018/7/26** | 2.630±0.240d | 2.273±0.211c | 0.357±0.046cd | 1.449±0.111d | 1.181±0.167a |
| SD9 | **2018/8/11** | 0.755±0.061e | 0.647±0.056d | 0.108±0.011e | 0.663±0.049e | 0.092±0.020e |

Note: SD1-SD9 are represented by different sowing dates from 2018/4/12 to 2018/8/11. Values represent means ± SE. Different lowercase letters within a column indicate significant differences among different sowing dates (ANOVA, LSD, *P* < 0.05). ***, *p* < 0.001. (The same below).

**Table S5.** Effects of sowing date on biomass allocation of different organs of *F. esculentum.*

| **Sowing dates** | | **Aboveground biomass allocation** | **Belowground biomass allocation** | **Vegetative biomass allocation** | **Reproductive biomass allocation** |
| --- | --- | --- | --- | --- | --- |
| SD1 | **2018/4/12** | 0.874±0.009bc | 0.126±0.009bc | 0.849±0.005a | 0.151±0.005c |
| SD2 | **2018/4/27** | 0.863±0.012bcd | 0.137±0.012abc | 0.860±0.006a | 0.140±0.006c |
| SD3 | **2018/5/12** | 0.872±0.011bc | 0.128±0.011bc | 0.855±0.005a | 0.145±0.005c |
| SD4 | **2018/5/27** | 0.884±0.007ab | 0.116±0.007cd | 0.849±0.006a | 0.151±0.006c |
| SD5 | **2018/6/11** | 0.859±0.014bcd | 0.141±0.014abc | 0.881±0.008a | 0.119±0.008c |
| SD6 | **2018/6/26** | 0.836±0.010d | 0.164±0.010a | 0.781±0.022b | 0.219±0.022b |
| SD7 | **2018/7/11** | 0.909±0.009a | 0.091±0.009d | 0.606±0.024c | 0.394±0.024a |
| SD8 | **2018/7/26** | 0.865±0.011bcd | 0.135±0.011abc | 0.587±0.029c | 0.413±0.029a |
| SD9 | **2018/8/11** | 0.851±0.012cd | 0.149±0.012ab | 0.889±0.014a | 0.111±0.014c |

**Table S6.** The allometric growth relationship of reproductive vs. vegetative biomass and belowground vs. aboveground biomass of *F. esculentum* among sowing treatment on the period of maximum reproductive biomass.

| Treatment | Traits | Allometric exponent | Allometric constant | *R*^2^ | *p* |
| --- | --- | --- | --- | --- | --- |
| SD1 | Reproductive vs. Vegetative | 1.585 | -1.163 | 0.639 | <0.05 |
| SD2 | Reproductive vs. Vegetative | 1.772 | -1.256 | 0.573 | <0.05 |
| SD3 | Reproductive vs. Vegetative | 1.789 | -1.244 | 0.707 | <0.05 |
| SD4 | Reproductive vs. Vegetative | 1.691 | -1.206 | 0.520 | <0.05 |
| SD5 | Reproductive vs. Vegetative | 1.577 | -1.126 | 0.436 | <0.05 |
| SD6 | Reproductive vs. Vegetative | 1.697 | -1.004 | 0.210 | <0.05 |
| SD7 | Reproductive vs. Vegetative | 1.684 | -0.699 | 0.127 | <0.05 |
| SD8 | Reproductive vs. Vegetative | 2.056 | -0.528 | 0.024 | <0.05 |
| SD9 | Reproductive vs. Vegetative | 1.462 | -0.741 | 0.400 | <0.05 |
| All | Reproductive vs. Vegetative | 1.306 | -0.901 | 0.413 | <0.05 |
| SD1 | Belowground vs. Aboveground vs.vvvvvs.aaaAboveground bbiomass bibiobbibiomass  Aboveground biomassABOVEGROUNDAAboveground vs.vvvvvs.aaaAboveground bbiomass bibiobbibiomass  Aboveground biomass | 1.278 | -1.036 | 0.551 | <0.05 |
| SD2 | Belowground vs. Aboveground vs.vvvvvs.aaaAboveground bbiomass bibiobbibiomass  Aboveground biomass | 1.461 | -1.160 | 0.507 | <0.05 |
| SD3 | Belowground vs. Aboveground vs.vvvvvs.aaaAboveground bbiomass bibiobbibiomass  Aboveground biomass | 1.373 | -1.065 | 0.436 | <0.05 |
| SD4 | Belowground vs. Aboveground vs.vvvvvs.aaaAboveground bbiomass bibiobbibiomass  Aboveground biomass | 1.608 | -1.083 | 0.389 | <0.05 |
| SD5 | Belowground vs. Aboveground vs.vvvvvs.aaaAboveground bbiomass bibiobbibiomass  Aboveground biomass | 1.898 | -1.238 | 0.230 | <0.05 |
| SD6 | Belowground vs. Aboveground vs.vvvvvs.aaaAboveground bbiomass bibiobbibiomass  Aboveground biomass | 1.507 | -1.128 | 0.318 | <0.05 |
| SD7 | Belowground vs. Aboveground vs.vvvvvs.aaaAboveground bbiomass bibiobbibiomass  Aboveground biomass | 1.520 | -1.171 | 0.434 | <0.05 |
| SD8 | Belowground vs. Aboveground vs.vvvvvs.aaaAboveground bbiomass bibiobbibiomass  Aboveground biomass | 1.530 | -1.027 | 0.597 | <0.05 |
| SD9 | Belowground vs. Aboveground vs.vvvvvs.aaaAboveground bbiomass bibiobbibiomass  Aboveground biomass | 1.404 | -0.767 | 0.464 | <0.05 |
| All | Belowground vs. Aboveground vs.vvvvvs.aaaAboveground bbiomass bibiobbibiomass  Aboveground biomass | 1.311 | -1.006 | 0.610 | <0.05 |

**Table S7.** The differences of allometric growth among sowing treatments (early, middle, late).

| Comparison of sowing treatments | Traits | Sowing treatments | Slope | Intercept | Slope homogeneity (p) | Shift in intercept (p) | Shift along common slope (p) |
| --- | --- | --- | --- | --- | --- | --- | --- |
| Early vs. Middle vs. Late | Reproductive vs. Vegetative | Early | 1.702 | -1.211 | **<0.05** | **<0.001** | **<0.001** |
|  |  | Middle | 1.572 | -1.097 |  |  |  |
|  |  | Late | 1.752 | -0.635 |  |  |  |
| Early vs. Middle | Reproductive vs. Vegetative | Early | 1.702 | -1.211 | **<0.001** | **<0.05** | **>0.05** |
|  |  | Middle | 1.572 | -1.097 |  |  |  |
| Early vs. Late | Reproductive vs. Vegetative | Early | 1.702 | -1.211 | 0.476 | **<0.001** | **<0.001** |
|  |  | Late | 1.752 | -0.635 |  |  |  |
| Middle vs. Late | Reproductive vs. Vegetative | Middle | 1.572 | -1.097 | **<0.05** | **<0.001** | **<0.001** |
|  |  | Late | 1.752 | -0.635 |  |  |  |
| Early vs. Middle vs. Late | Belowground vs. Aboveground | Early | 1.358 | -1.077 | **<0.001** | **<0.001** | **<0.001** |
|  |  | Middle | 1.712 | -1.17 |  |  |  |
|  |  | Late | 1.158 | -0.955 |  |  |  |
| Early vs. Middle | Belowground vs. Aboveground | Early | 1.358 | -1.077 | **<0.001** | **<0.001** | 0.507 |
|  |  | Middle | 1.712 | -1.17 |  |  |  |
| Early vs. Late | Belowground vs. Aboveground | Early | 1.358 | -1.077 | **<0.001** | **<0.001** | **<0.001** |
|  |  | Late | 1.158 | -0.955 |  |  |  |
| Middle vs. Late | Belowground vs. Aboveground | Middle | 1.712 | -1.17 | **<0.001** | **<0.05** | **<0.001** |
|  |  | Late | 1.158 | -0.955 |  |  |  |


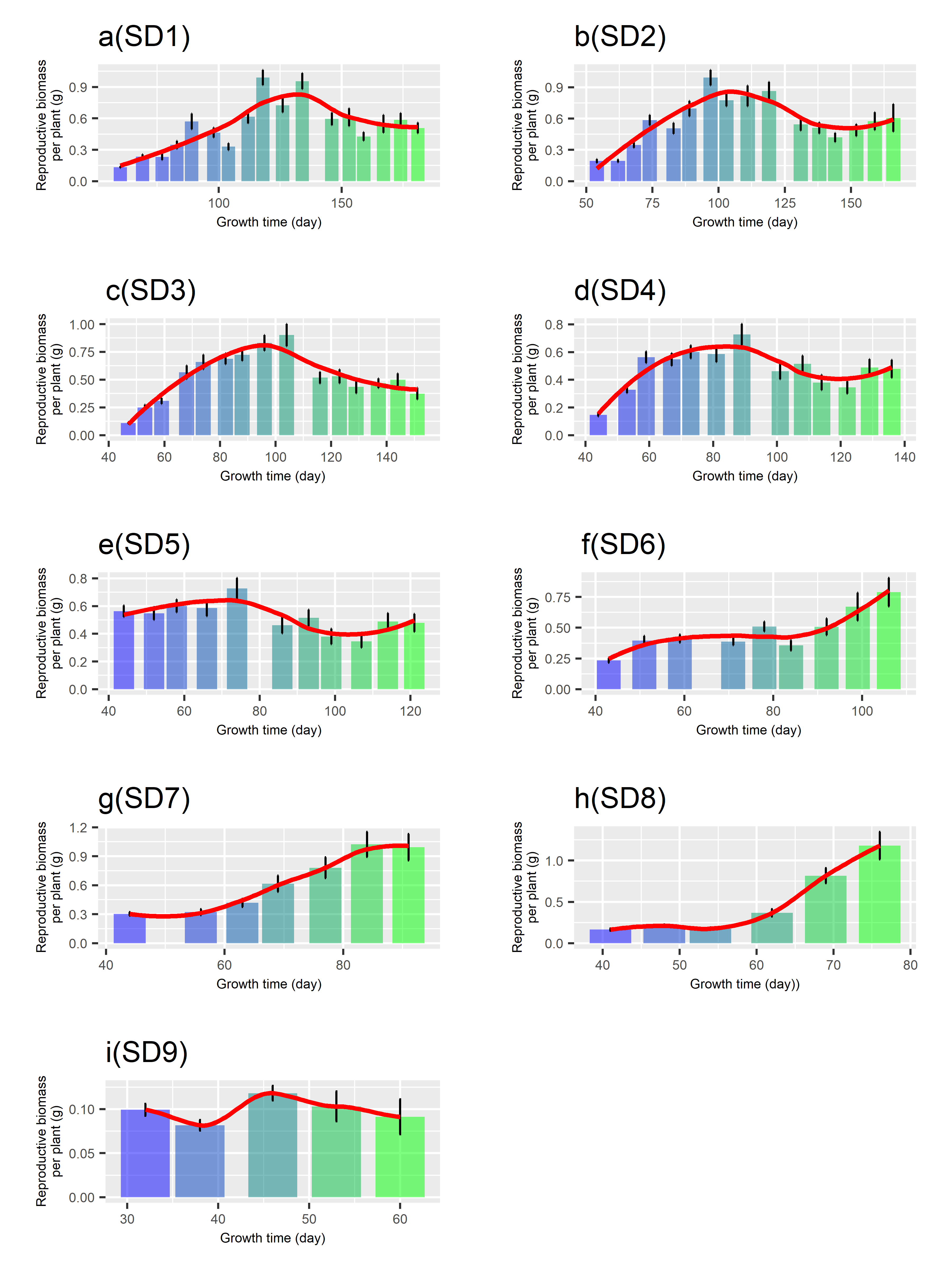


**Figure S1.** The reproductive biomass changed with the growth time under each sowing period.
